# Big Things Often Have Small Beginnings: A Review on the Development, Use and Value of Small and Big Corpora for Flemish Sign Language Linguistic Research

**DOI:** 10.3389/fpsyg.2021.779479

**Published:** 2022-01-05

**Authors:** Beatrijs Wille, Inez Beukeleers, Mieke Van Herreweghe, Myriam Vermeerbergen

**Affiliations:** ^1^Department of Linguistics, Ghent University, Ghent, Belgium; ^2^Department of Linguistics, KU Leuven, Leuven, Belgium; ^3^Department of Dutch and Afrikaans, Stellenbosch University, Stellenbosch, South Africa

**Keywords:** Flemish Sign Language, corpus linguistics, grammar, lexicography, sociolinguistics, automated sign language recognition, parent-child interactions, multifocal eye-tracking

## Abstract

In 1990, Vermeerbergen started the first larger-scale corpus study with (semi)spontaneous language data from adult signers on the morpho-syntactic aspects of Flemish Sign Language (VGT). After this, a number of lexicographic projects, including the collection of a 90-h corpus, led to the launch of the first online bilingual Dutch/VGT—VGT/Dutch dictionary in 2004. Since then, researchers have developed several corpora of variable sizes, with the greatest realization being the VGT Corpus. The main focus of this chapter is twofold. On the one hand the run-up to, the development and the use of the VGT Corpus will be discussed, while on the other hand smaller specific research corpora will be highlighted such as the corpus on early parent-child interaction and the multifocal eye-tracking corpus. The current chapter will discuss the research and community value of the corpora and future directions. Finally, it will elaborate on the need for corpus research, the associated advantages and disadvantages, and the obstacles faced in smaller deaf communities.

## Introduction

This article focuses on corpus developmental and documentary approaches to sign language research. It gives a major overview of the different Flemish Sign Language (VGT) projects utilizing various corpora in the past decades, with the main achievement being the VGT corpus (Van Herreweghe et al., 2015). The current state of affairs in Flanders and what we have learned from the development in sign language research will be discussed by looking at past, current and on-going projects. Prior to this, the chapter starts with a background description of Flemish Sign Language, the Flemish Deaf community and the main corpus project initiators. We are aware of the fact that in the past any set of data on which a linguistic analysis was performed was called a corpus but that with the advent of computer technology and corpus-based linguistics, use of the term “corpus” has become more and more restricted to any type of collection of texts in a machine-readable form. Nevertheless, we prefer to also label these older “datasets” corpora since we have the associated metadata and they were transcribed and/or annotated in machine readable text files—usually in Word -, be it not in an integrative way, i.e., not by means of a computer program that links the video data to transcription/annotation tiers as for instance the ELAN annotation software (Wittenburg et al., 2006).

After a unanimous vote, Flemish Sign Language was officially recognized by the Flemish Parliament in April 2006. The Flemish Government recognized VGT as a minority language used by the Deaf community in Flanders, for whom VGT possesses an identifying role (Vermeerbergen and Van Herreweghe, 2008; Van Herreweghe et al., 2016). Keeping in mind that not all deaf children acquire VGT and that not all signers are born deaf [e.g., interpreters, hearing children of deaf adults (CODAs)], Loots et al. (2003) estimated that there are 5,000–6,000 deaf Flemish Sign Language users. About 95% of them have hearing parents—i.e., who do not know how to sign at the time of their child’s birth. The vast majority of deaf people have acquired their sign language at the deaf school they attended.^1^ Hence, there are five distinct VGT regiolects corresponding to the areas around each Flemish deaf school which more or less coalesce with the Flemish provinces. Apart from regional variation, some gender-related differences—this inter-gender variation is especially visible in the older generations—can be noticed due to the existence of separate schools for boys and girls until the 1970s (De Weerdt et al., 2003; Vanhecke and De Weerdt, 2004; Jonckers, 2013). The Flemish Deaf community has formally rejected an imposed standardization and has instead openly stated to support and promote the ongoing spontaneous standardization process of the language (Van Herreweghe and Vermeerbergen, 2009). Therefore, inter- and intra-regional variation needs to be taken into account in every analysis of VGT.

The largest share of work focusing on the description of Flemish Sign Language has been continuously carried out by researchers now affiliated with Ghent University, KU Leuven and/or the Flemish Sign Language Center (VGTC). The VGTC, a non-profit organization, was founded in 1997. Later, in 2006, it was stipulated in the decree on the recognition of Flemish Sign Language that structural funding would be provided to the VGTC to develop as an independent center of expertise with respect to VGT (Van Herreweghe et al., 2016). Over the years, these three VGT research hubs have shared and supported each other in their own and in joint projects. However, the overall number of active researchers remains scarce.

## Small Corpora: Paving the Way Toward the Flemish Sign Language Corpus

This section discusses the development of early small corpora, the data collection process, and their results. It highlights the main studies on grammatical (2.1) and lexicographic research (2.2) since the start of VGT research in the 1990s until the establishment of the VGT Corpus in 2015.

### Grammatical Research From 1990 to 2015

#### The First Large Scale Study

When it comes to descriptive grammatical research, groundbreaking work was carried out by Vermeerbergen in the early 1990s, culminating in her PhD dissertation (1996). She collected and transcribed a corpus consisting of 6 h of spontaneous sign language data—4 h of dialogues and 2 h of monologs—produced by 10 (near-)native signers, at the time of the study between 30 and 83 years old. This spontaneous data was complemented with additional data, including narrative retellings as well as the elicitation of declarative (locative and non-locative) sentences from 14 informants (aged 22–86) based on the Volterra et al. (1984) picture task.

First, the full corpus was used to try and define VGT’s “basic word order,” i.e., the word order of simple declarative, active clauses. However, for VGT, a combination of one verb with two arguments (whether SVO or SOV) was less common than a combination of two clauses, each representing a subject/predicate structure (mostly as SVSV). The first part of the sentence constitutes the framework for the second part, which allows the combination to be seen as a topic/comment structure.

Second, with regard to Vermeerbergen’s other main research theme, i.e., the expression of the relationship between the verb and its arguments, her research shows that word order only plays a minor role (1996). Rather, VGT signers most often use one or more other linguistic mechanisms and constructions to indicate this relationship, including verb agreement, “classifier predicates,” the use of loci and pointing signs, role shifting (a.k.a. shifted attribution of expressive elements) and reference shifting, manual simultaneity and dominance reversals. Many of these mechanisms and constructions had already been described for other signed languages but had not yet been studied for Flemish Sign Language. In the following years, the main corpus, i.e., the 4 h of spontaneous dialogues, 2 h of monologs and 30 min of elicited narrative retelling, was used for several studies, e.g., on the use of space, non-manuals, classifiers and the productive lexicon, and simultaneity (e.g., Vermeerbergen, 1998, 2001, 2006; Vermeerbergen and Demey, 2007; Vermeerbergen et al., 2007a).

#### Other Grammatical Studies

Later, in the early 2000s, Van Herreweghe and Vermeerbergen (2003) jointly engaged in a new contrastive VGT—Dutch study focusing on reference tracking. The participants were asked to watch an animated cartoon, i.e., “Quatre à Voyager,” containing four main characters, all male (Faton and Theunen, 1983). The cartoon lasts about 7 min and does not contain any spoken interactions, nor subtitling. All the participants watched the cartoon twice and were asked to then narrate the story in written Dutch or in VGT. For the written Dutch stories there were 119 school-aged participants (these were collected by Van Herreweghe as part of her PhD research; Van Herreweghe, 1996). Eight signers participated in the production of the VGT narratives of whom six were native signers (four adolescents and two adults) and two were near-native signers (both adults). Van Herreweghe and Vermeerbergen (2003) showed that in VGT the protagonists could be referred to by means of full noun phrases (which was quite rare), pointing (in various ways), role and reference shifting, null arguments with (spatial) verb agreement or by simply deleting the subject (which is only possible in connected signing).

Shortly afterward the same researchers collaborated in a descriptive study on interrogatives and negatives in VGT (Van Herreweghe and Vermeerbergen, 2006). This time they used three small corpora: (1) parts of the corpus which was used by Vermeerbergen for her PhD, (2) nine versions of the “Quatre à Voyager” story (i.e., six versions by native signers and three by near-native signers), and (3) a game with two pairs of (near-)native signers who asked each other questions to which they expected a negative answer while none of them were allowed to simply sign YES or NO. In this way, more elaborate affirmative or negative utterances were elicited (this is a common children’s word game in Flanders).

In 2008, De Weerdt and Vermeerbergen further explored the expression of possession and existence in VGT. This study was part of a larger project coordinated by Ulrike Zeshan’s Sign Language Typology Group (Zeshan and Perniss, 2008). The researchers’ descriptions and the detailed number of examples were based on a questionnaire and additional data elicited from three near-native VGT-signers (De Weerdt and Vermeerbergen, 2008).

In the same year, the Flemish Sign Language Center initiated its first two research projects, namely on the topics of formation of plurals and the use of classifiers for the concepts “car,” “person,” and “bird” (Heyerick et al., 2011, 2014). The research data was primarily collected in the context of their study on plural formation (Heyerick and Van Braekevelt, 2008). The elicitation material used for these studies were 156 pictures of one object, two objects, multiple countable objects, and multiple uncountable objects (Kubusş, 2008; Zwitserlood et al., 2012). In addition to pictures, the participants were also exposed to two videos related to the researchers’ specific research questions, i.e., an advertising film for cars (duration 1:01) and the cartoon Birds (duration 2:38). Considering the (inter-)regional, gender and age variation, a total of 40 deaf VGT signers agreed to participate, i.e., 20 as active signers and 20 as interlocutors or recipients. This yielded 12 h of video data. Through this approach researchers were able to describe some mechanisms behind the formation of plurals and the formation of classifiers of those three specific referents, i.e., car, person, and bird. However, they stated that additional research is desirable and could include—among other suggestions—the recording and analysis of more spontaneous conversations (Heyerick et al., 2011).

Through cross-linguistic research, Vermeerbergen and Van Herreweghe also contributed to a further understanding of the degree of similarity between (un)related sign languages. The projects include the description of constituent order and verbal predicates in VGT and South African Sign Language (SASL) (Vermeerbergen et al., 2007b; Van Herreweghe and Vermeerbergen, 2012b). For this, a corpus driven by the Volterra et al. (1984) picture elicitation task was collected consisting of similar VGT and SASL-data, i.e., 4 signers per language producing the 18 sentences. The same type of data was collected for a cross-linguistic study including elicited declarative sentences from VGT, Irish Sign Language (ISL), and Auslan (Johnston et al., 2007). These studies showed that for non-locative sentences Flemish signers use both SVO and SOV order, with a preference for SOV in sentences with non-reversible arguments and SVO in sentences with reversible arguments. Whereas lexical verbs more often result in SVO order, productive “classifier predicates” appear in the final position. Furthermore, the analysis shows that, especially in the case of sentences with reversible arguments, Flemish signers often build more complex multi-clausal sentences or add elements such as an additional (main or light) verb resulting in split sentences, serial verb constructions or verb sandwiches.

### Lexicographic Research

From 1999 onward, several VGT lexicographic projects were conducted, which eventually led to the launch of the first online VGT/Dutch—Dutch/VGT dictionary in 2004 (see Van Herreweghe et al., 2004).^2^ This dictionary was based on the collected data of 30 informants, 6 per regional team. Each team consisted of deaf men and women between the ages of 20 and 50, all having a thorough proficiency in their regional VGT variety which they used in their daily lives. The informants had been educated at a deaf school and they all identified as being active members of the Flemish Deaf community. Within each regional team a deaf native VGT-signing moderator was appointed. These moderators received some prior training on eliciting and collecting the required data correctly. The full deaf team engaged in 6 thematic meetings, which eventually resulted in 90 h of recorded language data (see Vermeerbergen and Van Herreweghe, 2018 for a detailed overview of the thematic lists, the procedure, and the analysis). Since then, some studies have been conducted using the dictionary as their primary source of analysis. For instance, Demey’s doctoral dissertation is the sole extensive description of the phonological structure of VGT (Demey, 2005). This in-depth study includes a detailed phonetic transcription of 2,424 lexical signs, fingerspelling, and numbers. The results indicate that not only considerations of a phonological and phonetic nature are important when describing the form of signs, but also that the role of iconicity should not be underestimated. Further, contrastive research based on these transcriptions and analyses, and the phonological structure of the Sign Language of the Netherlands (NGT) demonstrates striking similarities, i.e., neighboring regions sharing the same spoken language, viz. Dutch (Vermeerbergen et al., 2013). Apart from a few frequency differences and additions to phonetic or semantic implementation rules, there is only little variation found among the two languages on a phonological level.

As part of her bachelor’s studies, De Putter (2016) used the VGT and NGT dictionaries to compare the signs for 100 basic concepts in both languages. She compared the manual parameters of the selected signs and found that the two languages have more signs classified as being different than similar or identical. The hand configuration proves to be the most language-specific parameter. The analysis of the non-manual parameter, however, reveals that the mouthing, derived from spoken Dutch in both languages, was identical in 85% of the cases and thus supports mutual intelligibility among these two sign languages.

## The Development of a Publicly Accessible Flemish Sign Language Corpus

The previously described early grammar and lexicographic projects have several things in common. Their results are based on the VGT productions of a limited number of deaf signers, recorded in a variety of different settings. Moreover, there is a rather small group of deaf VGT signers actively engaged within the Flemish Deaf community who have often been asked to participate in research. As a consequence, some of them regularly recur in several of these studies. In that way, the patterns that were identified in the different datasets might not be representative for the entire Flemish Deaf community. It should be noted that in most of these studies, the participating signers had to perform different elicitation tasks linked to a specific research question. Apart from Vermeerbergen (1996), these tasks often did not include free conversations, for instance. Moreover, these smaller corpora were never made publicly accessible since the informants had not been asked to consent to that. Consequently, the video data usually remained on different types of (analogous) videotapes or more recently on DVDs in the offices of the researchers. Only the transcriptions of the VGTC projects were carried out in the ELAN annotation software (Wittenburg et al., 2006), while the transcriptions of the other studies were mostly done in a separate text file. What’s more, the transcriptions were usually not complete since they only focused on the item to be studied. Also, the metadata collected were frequently of a different nature and therefore not always comparable. For all these reasons the demand for a representative corpus of Flemish Sign Language became more pressing.

Eventually, from 2012 to 2015 several VGT researchers collaborated in the development of an open access VGT Corpus (Van Herreweghe et al., 2015; Verstraete et al., 2015).^3^ The corpus was established to function as a core data source for any research effort aimed at analyzing VGT or comparing VGT with other (signed) languages. The machine-readable digital corpus of naturalistic and elicited Flemish Sign Language data includes more than 140 h of face-to-face interactional video data with a frame rate of 50 per second and a resolution of 960 by 544 pixels. Over the stretch of a number of years the research team collected data of 119 signers, i.e., native and near-native signers, men and women, with deaf parents and with hearing parents, between 12 and 91 years old. Overall, the corpus establishes a permanent and representative record of all VGT varieties, enabling the formulation of new observations on the use and structure of VGT. Moreover, it has a documentary function since the informants recount stories of their own schooldays, of activities in the Deaf community etc. The reference corpus of VGT can also be utilized for cross-linguistic purposes since part of the elicitation materials is used in research of other (sign) languages too (e.g., Sallandre et al., 2016).

Along with the collected metadata—personal background, patterns of language use, degree of bilingualism in VGT and Dutch, the corpus consists of elicited data, elicited and spontaneous narratives, conversational data as well as on-topic interviews (name sign, language attitudes, daily life during WWII etc.). In pairs, participants were asked to retell stories [e.g., “Frog where are you?” (Mayer, 1969) and “Quatre à Voyager” (Faton and Theunen, 1983)], to engage in free conversations, to sign the Volterra sentences (Volterra et al., 1984), to give road directions to the interlocutor, to explain the meaning behind their name signs, etc. The corpus is further enriched with ID glosses using the ELAN software (Wittenburg et al., 2006). The VGTC is currently working on a link with the VGT Signbank to incorporate ID-glossed signs into a lexical database and the dictionary.^4^ Since the start of the project 40 h of the data have been transcribed, i.e., for established lexical items with ID-glosses and for productive signing with a basic semantic annotation (both in written Dutch). As part of certain smaller research projects, some data in the corpus have been enriched with more detailed annotations on several aspects of the lexico-grammar of VGT. Several narratives have, for instance, been segmented and annotated for depicting signs and other types of depictive tokens, constructed action and role shifting, but also mouthings and eye gaze (e.g., Van Herreweghe and Vermeerbergen, 2012b; Beukeleers, 2015, 2016, 2020; Vaes, 2015; Pattyn, 2016; Beukeleers and Vermeerbergen, 2017; Braes, 2019; Van de Velde, 2019; De Vos, 2020; Goris, 2021). More recently, several researchers have also started annotating some of the conversational data in more depth while focusing on a certain aspect. As part of her bachelor’s paper, Aerts (2021) analyzed the data for PALM-UP and Jenard is—as part of an ongoing doctoral research project (MUST, 2020–2024)—analyzing the data for stance taking.

Finally, a small part of the data has been subtitled in Dutch—mostly explanations of people’s name signs—and therefore so far only these excerpts can be made available to and understood by a broader audience.

## Research Using the Flemish Sign Language Corpus

The VGT Corpus has frequently been consulted for educational and research purposes in recent years. This section provides a brief overview of studies that used the VGT Corpus—going from short-term projects in the context of Students’ research to larger PhD and long-term projects—and discusses the added value of these studies. We discuss how the corpus has been used to re-test previous claims in the VGT literature (4.1), to fill in particular research gaps (e.g., lexico-grammatical and sociolinguistic variation) (4.2), and as a source for the development of automatic sign language recognition software (4.3). Finally, the status of the VGT Corpus will be explained (4.4).

### Re-testing Previous Claims on a New Corpus and Documenting Language Change

As a consequence of natural language evolution, a constant revision of Flemish Sign Language linguistic research and outcomes is necessary (Vermeerbergen and Van Herreweghe, 2018). Moreover, as stated above, previous research was often carried out based on small and on-topic corpora with frequently recurring informants. Therefore, several researchers have repeated previous analyses on a new and more diverse corpus. In this way, they were not only able to re-test previous claims in the early VGT literature, but they could often also shed light onto language change in this particular language. Most reproduction studies were part of Students’ BA and MA papers, all supervised by at least one of the authors. Vandewalle (2016), for instance, used the corpus to re-test previous claims about the expression of negation in VGT reported on in Van Herreweghe and Vermeerbergen (2006, 2011). In his bachelor’s thesis, he analyzed data from 80 selected tasks produced by 82 participants, including both men and women across ages and regions. Results of the analysis of 599 tokens of negation show that manual negation signs do occur without an accompanied head movement. In this way, Vandewalle thus refutes the mandatory character of the head shake or negative hold in the expression of negation in VGT described in previous research (Van Herreweghe and Vermeerbergen, 2006, 2011). The “why” and “how” behind the findings are, however, still under investigation, as these questions are hard to answer on the basis of corpus data only.

In a similar vein, Braes (2019) echoed the work of Vermeerbergen (1996) to investigate word order and a possible evolution or change in VGT word order since the late 1990s. In the context of her bachelor’s thesis, she analyzed the Volterra declarative sentences (Volterra et al., 1984), taken from the VGT Corpus, which were produced by 6 informants (3 male and 3 female, 19–25 years old). Braes (2019) found that SVO still is the most commonly used sequence for sentences with reversible arguments. However, for sentences with non-reversible arguments no clear pattern stood out as SOV, SV and SVO were all found, indicating a large word order variation. Thus, Braes (2019) showed many similarities with the findings of Vermeerbergen (1996) and—however, based on fewer participants—carefully suggests that there is no distinct evolution or change in the word order of Flemish Sign Language.

### Filling in Some Research Gaps

As mentioned in the introduction, research on the lexico-grammar of VGT started only in the early 1990s. Moreover, there are not many researchers actively analyzing VGT. As a consequence, many aspects of the language have not yet been studied (in great detail). Therefore, some researchers have been using the corpus to address some research gaps. In this way, the VGT Corpus has paved the way for some initial studies on, for instance, the influence of elicitation materials on the use of signing space (Beukeleers, 2015, 2016; Beukeleers and Vermeerbergen, 2017), the functions of the sign TO-HAVE (Sampson, 2016), repetitions (Notarrigo et al., 2016), mouthings and mouth gestures (Pattyn, 2016; Van de Velde, 2019; De Vos, 2020), constructed action (Beukeleers, 2020; Goris, 2021), and the functions of PALM-UP (Aerts, 2021).

As researchers have transcribed and partly annotated the corpus data in ELAN, the corpus is developing into a full machine-readable corpus, which not only simplifies the analysis of the data, but also facilitates the exportation of the data to other software. As a result, most recent studies—including the studies mentioned above—now report on the exact frequencies behind the patterns and more quantitative research has been initiated. In this regard, the development of a machine-readable corpus of VGT language use has enabled, for instance, some first studies on lexical frequency (Sampson, 2017; Bruynseraede, 2018). Analyzing 8 and 20 narratives, respectively, Master students Sampson (2017) and Bruynseraede (2018) found that fully lexical signs, i.e., established form-meaning pairings, are the most frequently used signs in the data. These conventionalized forms are followed by signs from the productive lexicon, i.e., classifier constructions and constructed action. Pointing signs and gestures occur less frequently.

Quantitative studies like the ones above are not only highly relevant for a more thorough and comprehensive description of the lexico-grammar of VGT, but also for the field of applied linguistics (Johnston, 2010). Based on the frequencies of signs and formulations, teachers can provide L2 learners with the most frequent vocabulary and formulations first. Less frequent signs and formulations can then be integrated later in the L2 training program. In this way, insights from studies based on the VGT Corpus can—in the long run—be integrated in the curriculum of VGT training programs.

When reviewing the literature on Flemish Sign Language, it also becomes apparent that since the development of the VGT Corpus, more researchers have carried out sociolinguistic research. Vermeerbergen and Van Herreweghe (2013), for instance, analyze 12 retellings of “The Horse Story” (Hickmann, 2003). They selected participants from 3 generations of signers: 17–25 years old, 40–50 years old, and + 75 years old. For each generation, Van Herreweghe and Vermeerbergen selected 2 native and 2 non-native signers. Results reveal age-related variation in the choice for a particular sign type. Whereas older signers (+75 years old) use elements of the productive lexicon, i.e., classifier constructions and bodily enactment, more frequently, younger signers rely more heavily on the frozen lexicon, i.e., on established form-meaning pairings.

Other topic-specific sociolinguistic research on VGT, carried out by BA and MA students, includes a study on internal and external linguistic factors influencing the variation of the two-handed sign COW (De Putter, 2019), on age-based lexical variation in the choice for signs that refer to the days of the week (Swennen, 2018), on register variation (Vandewalle, 2018), on gender-based variation in simultaneous constructions (Van Deuren, 2019), and on regional variation in the expression of negation (Hollevoet, 2021).

### The Flemish Sign Language Corpus as a Source for the Development of Automatic Sign Language Recognition Software

Finally, the VGT Corpus is also being used in sign language recognition studies (SLR). Several doctoral researchers have used the data to develop Automatic Sign Language Recognition software. Pigou (2018), for instance, focused on deep neural networks. To overcome the relatively small size of the already transcribed sections of the VGT Corpus at the time, other data such as transcribed interpreted Flemish TV news broadcasts and the NGT Corpus (Sign Language of the Netherlands; Crasborn et al., 2008)—which included more annotated data—were also included in the study (Pigou, 2018). De Coster’s PhD research focuses on video transformer networks with hand cropping and pose flow (De Coster et al., 2021). As there are still many open research questions regarding SLR, two new promising interdisciplinary research projects have been launched since. These European SLR projects—that is SignON (European Commission, 2020b) and EASIER (European Commission, 2020a) funded by Horizon 2020—aim to facilitate the exchange of information among hearing and deaf individuals across Europe, each including several spoken and signed languages. In this light, the VGT Corpus is not only used for the purpose of theoretical language description, but also as a data source for the development of, for instance, automatic translation of VGT into written/spoken Dutch.

### The Current Status of the Flemish Sign Language Corpus

In sum, this section has shed light on the added value of the VGT Corpus in the study of Flemish Sign Language. In doing so, we have shown that the corpus has allowed researchers to re-test previous claims in the early literature on new (and often more) data, to fill in particular gaps in our knowledge about the structure of VGT, to document sociolinguistic variation and in the development of automatic sign language recognition and translation software. Although the corpus has proven to be an important asset in the study of VGT in the last 6 years, it should be noted that the development of the corpus itself is in many ways still in its infancy. Due to the limited number of researchers working on VGT large parts of the corpus have not been transcribed and/or annotated (in great detail). Consequently, the size of the datasets of current studies on VGT still remains rather small and thus many of the findings and conclusions in the studies reported on above, are still rather preliminary. We will return to this in the discussion of this paper (see section “Discussion”).

## Beyond the Flemish Sign Language Corpus

In the context of several doctoral projects, two small corpora have been developed over the years focusing on other aspects of Flemish Sign Language. The first corpus is based on early dyadic parent-child interactions (Loots, 1999; Mouvet, 2013) and a second one, the multifocal eye-tracking corpus, combines the use of static cameras with mobile eye-tracking devices to study the role of eye gaze in triadic VGT interactions (Beukeleers, 2020).

### A Corpus for the Study of Early Parent-Child Interactions

The first corpus, based on early parent-child interactions, calls on the data collection of the doctoral projects of Loots (1999) and of Mouvet and Matthijs (2010–2013).^5^ This full corpus—compiled with data collected at two different points in time—is innovative for VGT in many ways. It includes an age group not studied before, interlocutors of different ages, different language backgrounds, spontaneous conversations etc. The corpus includes infants aged 6 months up to 2 years old. The video recordings contain parent—child interactions of when the children were 6, 9, 12, 18, and 24 months old. The researchers aimed to fully reflect the heterogeneity of the general population of deaf children, e.g., language background and auditory support. Overall, 90–95% of the deaf children are raised in a hearing family with no prior knowledge on deafness or the visual modality. Therefore, the corpus includes many hearing parents and a handful of deaf parents. All children were deaf or hard of hearing (DHH). Researchers of both projects made sure that all regions in Flanders were represented in their corpus. The initial corpus consisted of 20 parent-child dyads, with 13 originating from hearing families and 7 from deaf families. Data collection included mothers and fathers interacting with their DHH child. These interactions were recorded in a therapeutic setting. Mouvet and Matthijs based their work on the early interactions of DHH children in 12 hearing families and 1 deaf family. All interactions were recorded in the home setting. Depending on children’s age and concentration span, and the initial research purposes these recordings vary from 7 to 45 min of dyadic interactions.

The corpus has been analyzed by Loots (1999); Mouvet (2013), Matthijs (2018), and Wille (2018) for different research purposes and has given us a first and profound insight in the early interactions and language environment of deaf infants. Loots, for instance, concluded that parents who do not communicate visually and in a sequential manner with their deaf child stagnate in the transition from existential to symbolic intersubjectivity between the ages of 18 and 24 months (Loots and Devisé, 2003a; Loots et al., 2005a). Mouvet (2013) later showed that the functional development of the deaf children of hearing parents in her study was clearly delayed, showing individual developmental patterns with respect to language(s) and/or modality(ies). She continues by stating that these deaf children, regardless of the type of auditory support, do not perform on par with their hearing peers nor is their signed lexicon comparable to that of their deaf native signing peers, potentially resulting in semilingualism. In addition, Wille et al. (2018), who analyzed data collected by Mouvet and Matthijs, were the first to describe the VGT development of a deaf child growing up in a deaf signing family, with respect to dyadic face-to-face interactions. Along with a more extensive corpus analysis, using data from Loots (1999), and Mouvet and Matthijs (2010–2013), this research provided the basis for the development of early interaction guidelines for professionals; including deaf children’s early visual milestones (Wille et al., 2019, 2020a,b). The studies above, emphasize the benefits of a bimodal bilingual approach which can facilitate language development and may form a supportive basis for the children’s full language potential.

The corpora further also formed the base of research on early communication strategies used by deaf and hearing parents (mothers and fathers) in interaction with their DHH children. Through their analyses, these researchers have been able to show the influence of parents’ hearing status, gender, and language choice on their use of these strategies (Loots and Devisé, 2003b; Loots et al., 2005b; Wille et al., 2019). In line with these findings, Matthijs (2018) highlights the role of *good quality* parental communication strategies within deaf children’s development of intersubjectivity in mother-child interactions. The researchers above all highlight the insufficient visual support hearing parents receive and indicate that deaf parents can be seen as role models for all hearing parents when it comes to efficient early communication with a deaf child, independent of children’s auditory support and parental language choices.

### A Multifocal Eye-Tracking Corpus

Most recently, a new corpus for the study of the role of eye gaze in VGT interactions was developed. Beukeleers PhD project (2020) investigated the role of eye gaze in VGT interactions focusing on its functions regarding turn management and its various functions in the lexico-grammar. When reviewing the sign language linguistics literature, it became apparent that many topics related to the functions of eye gaze in interaction management have not been studied in great depth. Moreover, most of the existing studies analyzed only a limited amount of data (i.e., 2–13 dialogues, see Baker, 1977; Lackner, 2009) and focused merely on the analysis of dialogues, where there might be less competition for the floor compared to triadic and multi-party interactions. Moreover, most researchers have mainly analyzed these functions of eye gaze in video data that were only recorded with (a) static camera(s). It is, however, not straightforward to analyze interlocutors’ gaze behavior in this type of datasets, because the videos do not allow researchers to determine the exact point of fixation, i.e., to determine where the participant is exactly looking at. Researchers rather have to estimate participants’ gaze directions by relying on their head and eye movements. To overcome these limitations, Beukeleers et al. (2020) opted to build a new corpus using a multifocal eye-tracking approach (see Brône and Oben, 2015). This corpus contains 10 unscripted triadic VGT interactions, including both conversations on a topic of the participants’ choice and brainstorm sessions on a given topic. Each conversation lasted about 20 min and in total 2 h55 of data were collected. Altogether, 12 fluent signers—male and female—have engaged in the interactions. The participants come from the 5 different regions in Flanders and vary in age (22–75 years old). Participants were grouped per 3 and seated in a triangle to ensure that they had equal visual access to both co-participants. The interactions were recorded with 3 static cameras and participants were also equipped with mobile eye-tracking devices which record the environment from each participant’s perspective and simultaneously measured their eye movements (Beukeleers et al., 2020; for more technical details see Beukeleers, 2020).

This dataset has made it possible for researchers to analyze unaddressed participants’ gaze behavior during question-response sequences in triadic interactions. By timing the unaddressed participant’s gaze shift in relation to the end of the question of the signer and to the beginning of the next signer’s response, Beukeleers (2020) and Beukeleers et al. (2020) have used ratified participants’ gaze shifts as an empirical measure of anticipation in face-to-face signed discourse. In this way, they have contributed to a better understanding of turn processing and anticipation in interaction (Beukeleers, 2020; Beukeleers et al., 2020). Moreover, the eye-tracking data allow for an analysis of signers’ gaze behavior in the construal of multimodal depictions and in turn management, i.e., two functions that have gained only little scholarly attention within the field of sign language linguistics. Beukeleers (2020) has, for instance, shown how these functions can co-occur and compete in spontaneous conversation. Whereas previous studies have often assumed that signers systematically look at depictions when creating them, Beukeleers (2020) has shown that gaze patterns rather differ according to, for instance, the type of turn (depictions in a question vs. non-question turn) and the position of the depiction in the turn (e.g., turn-medial vs. turn-final position). Hence, she argues that eye gaze plays a prominent role at different levels of social interaction and that its function is dependent on the context it occurs in and the social action that is being performed (see also Rossano (2012) and Kendrick and Holler (2017) for spoken Italian and English, respectively). Parallel corpora are available, thus it is not only possible to further explore other social functions of eye gaze and their interplay in VGT discourse, but also to initiate comparative studies on the use of eye gaze, and even more broadly, the multimodal nature of face-to-face interaction and the tight integration of gesture and language.^6^

## Discussion

This section contains a critical reflection on the need for and the use of corpus research. It will elaborate on the associated advantages and disadvantages of corpus development, and the obstacles faced in smaller deaf communities.

Corpus linguistics goes hand in hand with the possibilities offered by more and more advanced computer technology. These advancements have allowed us to store large amounts of digital data, and to develop time-aligned annotation software such as ELAN.^7^ Indeed, Johnston (2009: 18) argues: “Corpus linguistics is based on the assumption that processing large amounts of annotated texts can reveal patterns of language use and structure not available to lay user intuitions or even to export detailed linguistic analysis of particular texts.” In corpus linguistics, “quantitative analysis goes hand in hand with qualitative analysis” (Leech, 2000, p. 49). The same obviously holds for sign language corpora. The research value of the collected authentic language data for VGT has proven to be undeniably high. Since the 1990s individuals’ intuitions have been gradually supplemented by concrete linguistic evidence from smaller scaled specific corpora. The more recently developed and (partly) searchable VGT Corpus allows for a large-scale approach and in-depth analysis of almost all language patterns. Through this new data collection and analyses, initial statements have been and will be rejected, confirmed, or broadened. New statements have also been formulated on targeted questions linked to region, gender, age, register, and task variation. Nevertheless, even though more and more signed language corpora emerge, we still feel that for certain types of research, it is recommended to adopt an integrated approach in sign linguistic research as was stated in Van Herreweghe and Vermeerbergen (2012a, p. 1033). Such an approach could involve analyzing corpus data (quantitatively and qualitatively), making hypotheses on the basis of this analysis and checking these hypotheses against native signers’ intuitions. The latter comes with risks as Pateman (1987, p. 100), for instance, argued: “it is clear and admitted that intuitions of grammaticality are liable to all kinds of interference ‘on the way up’ to the level at which they are given as responses to questions. In particular, they are liable to interference from social judgments of linguistic acceptability.” However, we feel that the combination of corpus data analyses and subsequently tapping into native signers’ intuitions can to a large extent mitigate this kind of interference. What is more, certain rare constructions may not occur in the corpora at hand and negative information (i.e., ungrammatical or unacceptable utterances) cannot be inferred from corpora. That is why native signers’ intuitions, grammaticality judgment tasks, and experimental studies should remain complementary to corpus research.

The open access nature of the videos included in the VGT Corpus allows all of us to witness how language is used in different contexts by different signers, as it includes many different tasks and is balanced for age, gender, and region. This data should then be accompanied by transcriptions which convert it into a machine-readable and searchable corpus, to facilitate more complex analyses, instead of a static archive. Researchers working on the VGT Corpus project have so far mainly focused on the transcription of the manual production, i.e., of established and productive signs. Fully annotating a signed corpus is extremely labor intensive and time consuming. Crasborn (2014) mentions that “glossing in annotation software can take as much as 200 times real time to do consistently—assuming there is already a full lexicon with ID-glosses available for reference,” such as a Signbank. In the future this might change due to the current efforts toward automatic sign recognition (see section “The Flemish Sign Language Corpus as a Source for the Development of Automatic Sign Language Recognition software”). At the moment the most frequently occurring signs in the VGT corpus, for instance, can be transcribed with a relatively accurate automatic sign recognition tool using a drop-down menu with five suggestions of the most likely automatically recognized sign (Pigou, 2018; De Coster et al., 2021). It is expected that quite a lot of progress can still be made in this area and VGT annotators impatiently look forward to this. After several years of varying work intensity, the transcription and annotation process is still not finished for the VGT Corpus, so that researchers continuously transcribing and annotating the collected data are necessarily engaged in long-term work. Many countries face a similar slow annotation process, not only because of the technical aspects related to annotation, but also because of financial reasons. It is often impossible, once the video data for a corpus is collected, to find additional funding to add the much needed annotations to the corpus. As a result, annotations are often done in the context of student theses or doctoral projects. This precisely contradicts some of the initial aims of building a corpus, i.e., direct use and facilitation of (complex) linguistic research with the help of a fully machine-readable corpus, where researchers would not have to put so much time into annotation anymore. Consequently, the size of the datasets that have been annotated and analyzed in light of these projects still remains rather small and mostly topic specific. Thus, large parts of the VGT Corpus are still not machine-readable. Additionally, most of the studies that use the VGT Corpus data consider analyses of retellings of narratives and do not include conversational data (with the exception of Notarrigo et al., 2016; Aerts, 2021). These limitations imply that many of the findings and conclusions in the studies reported on above (see sections “Re-testing Previous Claims on a New Corpus and Documenting Language Change and Filling in Some Research Gaps”), are to a certain extent preliminary and they thus should be ratified with analyses of larger, and more representative samples of VGT data. This is exactly the main reason why the VGT Corpus (like many other sign language corpora) was built.

However, once (part of) a corpus has been transcribed (and annotated) its value is undeniably great and comes with many advantages such as its representativity, the readily availability of the language data, and the possibility of carrying out new, large frequency and variation studies. This is, for instance, how we build a better understanding of early Flemish Sign Language acquisition, language access to deaf children and the tight integration of early gestures and sign language development (see section “A Corpus for the Study of Early Parent-Child Interactions”). The most recent corpus—including innovative eye tracking—has also provided an insight in online turn processing, the construction of depictions and the role of eye gaze (see section “A Multifocal Eye-Tracking Corpus”). The descriptions of linguistic patterns can then be converted into detailed language and teaching materials for prospective linguists, interpreters, speech- and language therapists, and teachers.

Within the deaf community, studies based on corpus data can also support the creation of a broader understanding and greater awareness of Flemish Sign Language. Research can further be integrated in education, social valorization projects and on-topic workshops to continuously disseminate new research findings. In this regard, it remains important to add new data to corpora—such as the VGT Corpus—on a regular basis, especially from younger signers who were too young during earlier recording moments. A corpus is a documentation of language use, but one cannot lose sight of new language evolutions. The past years, researchers have described several changes related to VGT use and practices, e.g., the recognition of VGT, societal and educational values of VGT, access to (tertiary) education, technological advances, internationalization (Van Herreweghe et al., 2016). It seems that VGT is going through an accelerated development, involving an exponential growth of the lexicon, the development of a formal vs. informal register, and the introduction of “new” signs and structures (e.g., the VGT sign APPARENTLY; Vermeerbergen, 2020). Therefore, researchers should proceed to collect new data and thus constantly maintain and expand the database so that research on and the teaching of sign language structures, and the documentation of signs used in the language continue to evolve with the language and its context. So, it is obvious that a solid corpus requires constant attention and a hands-on active approach.

## Author Contributions

BW, MVH, and MV conceived and planned the project. BW took the lead in writing the draft manuscript. BW and IB further shaped the presented ideas. All authors provided critical feedback and contributed to the final version of the manuscript.

## Conflict of Interest

The authors declare that the research was conducted in the absence of any commercial or financial relationships that could be construed as a potential conflict of interest.

## Publisher’s Note

All claims expressed in this article are solely those of the authors and do not necessarily represent those of their affiliated organizations, or those of the publisher, the editors and the reviewers. Any product that may be evaluated in this article, or claim that may be made by its manufacturer, is not guaranteed or endorsed by the publisher.

## Footnotes

1 This might, however, be different for the current generation of deaf children. These pupils integrate more regularly into mainstream education compared to the older generations.

2
https://woordenboek.vlaamsegebarentaal.be

3
http://www.corpusvgt.be

4 The VGT Signbank—part of the Global Signbank (Crasborn et al., 2018)—has currently about 20 000 entries of which 9959 are already accessible through the online dictionary. Every entry in the dataset receives a unique code referring to the corresponding ID gloss.

5 Mouvet and Matthijs collected their data together during the period 2010–2013, resulting in two PhD dissertations (respectively, in 2013 and 2018).

6 See, for instance, Vranjes (2018) for a multifocal eye-tracking corpus of interpreter-mediated spoken Dutch-Russian conversations, Jehoul (2019) for a multifocal eye-tracking corpus of spoken Dutch conversations and Vandemoortele (2020) for a multifocal eye-tracking corpus of musical interactions.

7
https://archive.mpi.nl/tla/elan
